# Mechanical properties of bulk Sylgard 184 and its extension with silicone oil

**DOI:** 10.1038/s41598-021-98694-2

**Published:** 2021-09-27

**Authors:** R. Moučka, M. Sedlačík, J. Osička, V. Pata

**Affiliations:** 1 Centre of Polymer Systems, University Institute, Tomas Bata University in Zlín, Trida T. Bati 5678, 760 01 Zlín, Czech Republic; 2 Polymer Centre, Faculty of Technology, Tomas Bata University in Zlín, Vavrečkova 275, 760 01 Zlín, Czech Republic; 3 Department of Production Engineering, Faculty of Technology, Tomas Bata University in Zlín, Vavrečkova 275, 760 01 Zlín, Czech Republic

**Keywords:** Engineering, Materials science

## Abstract

Due to its simple curing and very good mechanical properties, Sylgard 184 belongs to the most widely and frequently used silicones in many industrial applications such as microfluidics and microengineering. On top of that its mechanical properties are further controllable through the curing temperature, which may vary from ambient temperature up to 200 °C; the lower the curing temperature the lower the mechanical properties (Johnston et al. in J Micromech Microeng 24:7, 2014. 10.1088/0960-1317/24/3/035017). However, certain specialised application may require even a softer binder than the low curing temperature allows for. In this study we show that this softening can be achieved with the addition of silicone oil into the Sylgard 184 system. To this end a series of Sylgard 184 samples with varying silicone oil concentrations were prepared and tested (tensile test, rotational rheometer) in order to determine how curing temperature and silicone oil content affect mechanical properties. Curing reaction of the polymer system was found to observe 2nd order kinetics in all cases, regardless the oil concentration used. The results suggest that within the tested concentration range the silicone oil addition can be used to soften commercial silicone Sylgard 184.

## Introduction

Due to their simple fabrication, good transparency, chemical inertness, biocompatibility, and elasticity with decent mechanical properties, silicone elastomers, namely polydimethylsiloxane (PDMS), have quickly become indispensable in many applications. They are used in stretchable wearable electronics^1^, biomedicine^2,3^, microelectromechanical systems (MEMS)^4^, microfluidic chips^5^, in biomaterials for studying cell mechanobiology^6^ and lately also in the field of magnetorheology^7,8^. One field where PDMS excel/dominate is soft-lithography^9,10^, a technique that allows rapid prototyping of microfluidic devices^11–13^, as they offer a mix of low toxicity, biocompatibility, mechanical flexibility and durability, low cost.

Even though for many applications their inherent mechanical properties^14^, namely elastic modulus, are considered rather soft, and thus these would prefer slightly harder binder^15^, there are other fields (such as microfluidic, micro electro systems or magnetorheology) which benefit greatly from high flexibility and softness of Sylgard 184. Magnetorheology, for instance, requires a polymer binder which would be in an off-state quite soft and thus after application of an external magnetic field record a significant reinforcing effect due to fixation of magnetic particles’ positions^16,17^.

There are several approaches to how one can soften thermosets^18^. Generally, one needs to increase the distance between cross-links, which can be achieved, for instance, through using bottle-brush polymers^19^, by choice of polymerization strategy^20^, change of molecular weight^21^, decreasing concentration of reactive groups^22^, addition of plasticizer^23^, increasing dangling chains^24^ or changing crosslinker functionality^25^. This can be in theory accomplished by changing the ratio between a prepolymer and a curing agent. However, commercial systems tend to yield in such a case not fully cured system which exhibits negative effects (e.g. extreme stickiness). Thus, it is often better to try increasing the distance of polymer network nodes by incorporating low molecular substance with a good affinity towards prepolymer and having the same functional groups that allow for curing with the particular curing agent. There is no or very little previous research using this approach, thus we have decided to add a silicone oil to the reaction components of investigated PDMS, i.e. Sylgard 184, which can also often serve as an additive improving inherent viscosity, e.g. for better particles’ dispersing^26^.

The effects of the oil addition and curing temperature used are studied in order to determine optimal settings, for which sufficient softening (observed through elastic modulus decrease) of Sylgard 184 takes place without occurrence of negative phenomena such separation of silicone oil from Sylgard 184 composition or considerable deterioration of strength of the polymer. We present an experimental study delving into the effect of curing temperature on final mechanical properties of Sylgard 184, a matrix widely used in industrial applications. Also we notice how this polymer system can be further “softened” by extension with silicone oil for the purpose of applications requiring highly elastic soft polymeric matrix/binder with simple method of preparation.

## Experimental

Effect of two parameters, curing temperature and silicone oil concentration, on mechanical properties (Young’s elastic and shear moduli) was studied, to which end corresponding sets of samples were prepared and subsequently tested.

### Materials

A two-component kit (a prepolymer and a curing agent default mixing weight ratio 10:1) of commercially available silicone elastomer Sylgard 184 (Dow Corning, USA) was used as a testing matrix/binder. As a “softener” silicone oil (Lukosiol M200, Chemical Works Kolín, Czech Republic, viscosity *η* = 194 mPa s) was employed.

### Sample preparation

Samples for tensile test were prepared by mixing silicone prepolymer and a curing agent in the (manufacturer recommended) wt. ratio of 10:1 and adding a silicone oil in corresponding concentration, namely 0, 10, 20 or 30 wt.% related to the prepolymer only. The mixture was first stirred (10 min at 100 rpm by vacuum mixer) then carefully cast into a rectangular mould (120 × 120 × 2 mm) in order to prevent introduction of air bubbles. Each of these four variously concentrated systems was cured at three different temperatures for time period recommended by Dow Corning, which was corrected for heat propagation effects determined separately earlier (Table 1). This prior determination involved measurement of target temperature time delay in the centre of the mould and basically came very close to values stated in^27^.

**Table 1:** Curing parameters.

Sample	Curing temperature (°C)	Curing time (h:min)
S_25	25	48:00
S_100	100	0:40
S_120	120	0:30
S_150	150	0:20

Thus a set of 12 samples, varying either in silicone oil content or curing temperature, was obtained. Identical composition was used also for rheological tests however the mixture was not cast but directly loaded into the rheometer in this case.

### Mechanical testing

Mechanical properties of investigated silicone systems were studied via a tensile test and shearing on a rotational rheometer.

Tensile test was carried out according to ASTM D412 using Testometric MT350-5CT on the “dogbone” shaped samples 80 mm long and 10 mm wide with thickness of approximately 2 mm (exact values measured individually), which were cut out from rectangular plates prepared earlier. The elongation rate was set to 254 mm min^–1^. Values of Young’s modulus (*E*) in elongation were calculated from the linear part of the stress (*σ*) versus strain (*ε*) dependence recorded below 10% of *ε*. All samples were measured 48 h after their preparation and proper conditioning at temperature of 20 °C and relative humidity of 35%.

Shear modulus was obtained from oscillatory experiments using a rotational rheometer (Bohlin Gemini, Malvern Instruments, UK) with parallel plate geometry 20 mm in diameter and a gap of 1 mm. Constant frequency of 1 Hz and strain of 0.0004 (within a range of linear viscoelastic region) were applied during the experiment. Storage modulus, *G*′, evolution was being recorded continuously in time as the sample was cured in the rheometer at given temperature for the time period two times longer than that recommended by Dow Corning to make sure the samples were fully crosslinked. To simulate real working conditions all samples were heated from the ambient temperature (20 °C) up to the curing temperature in times given in Table 2. Thus, curing was monitored in all its stages.

**Table 2:** Curing temperatures and times necessary to reach them in crosslinking rheological tests.

Curing temperature (°C)	Heating time (s)
100	90
120	105
150	120

## Results and discussion

**Figure 1:**
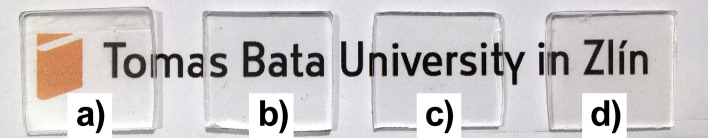
Digital photograph of the prepared silicone elastomers with the portion of Sylgard 184:silicone oil. (**a**) 10:0, (**b**) 9:1, (**c**) 8:2, and (**d**) 7:3.

The objective of the study was to determine impact of curing temperature and addition of silicone oil on mechanical properties of silicone elastomer. Figure 1 shows a macroscopic view of the fabricated elastomers cured at 100 °C differing in silicone oil content. All the samples are evidently transparent and without any changes of colour even when thinned by silicone oil addition when placed on a flat surface.

### Impact of curing temperature

**Figure 2:**
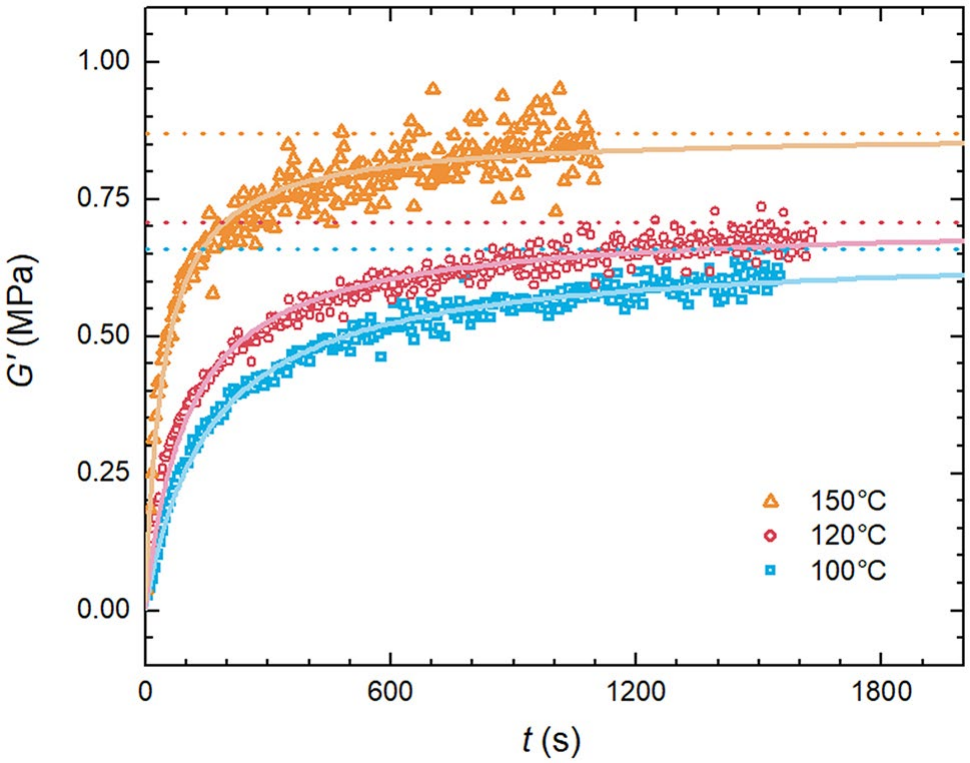
Impact of curing temperature on the kinetics of Sylgard 184 crosslinking (curing).

First the sole impact of curing temperature on Sylgard 184 with no additional silicone oil was investigated. Curing reaction of Sylgard 184 was monitored using rotational rheometer heated to given temperature filled with a mixture of the prepolymer and curing agent in the mixing ratio 10:1 (as recommended by Dow Corning). Time evolution of a *G*′ of the mixture was measured. Thus obtained dependence shows monotonous increase of *G*′ with time as the curing process takes place followed by a concavely shaped curved asymptotically approaching its maximum, where all curing agent is used up and Sylgard 184 is fully cured (Fig. 2). As can be seen the recorded curves for all three temperatures have exponential character and are well approximated by second order kinetics (as Sylgard 184 is a two component reaction system) model with a following differential equation:

1 $$ \frac{d\alpha }{{dt}} = kc_{0} \left( {1 - \alpha } \right)^{2} $$

where *k* (M^–1^ s^–1^) is a rate constant and *c*_0_ (M) is an initial concentration, *α* (–) is conversion defined as:

2 $$ \alpha = \frac{{G_{{\text{t}}}^{^{\prime}} - G_{0}^{^{\prime}} }}{{G_{\infty }^{^{\prime}} - G_{0}^{^{\prime}} }} $$

where *G*′_t_ (Pa) is shear modulus at time *t *(s), *G*′_0_ (Pa) starting modulus, *G*′_∞_ (Pa) fully cured Sylgard 184 modulus.

Equation (1) has following solution:

3 $$ \alpha \left( t \right) = 1 - \frac{1}{{kc_{0} t + 1}}, $$

which was used to fit the experimental data and enabled extraction of the rate constant *k* and the maximum shear modulus *G*_∞_ (at fully cured state—marked by dotted lines in Fig. 2).

Thus, one can see that usage of higher curing temperature not only shortens curing process (see *k* values in Table 3) but also yields samples with higher shear modulus^27^.

**Table 3:** Kinetics parameters.

Sample	Rate constant, *k* (s^–1^)	*G′*_∞/150 °C norm./_
S_25	–	–
S_100	0.0079	0.76
S_120	0.0097	0.81
S_150	0.0209	1

Mechanical properties of elastomer are ultimately given by the structure of its three-dimensional polymer network, which depends on functionality of crosslinks, entanglements and defects but probably most significantly/importantly on the molecular weight of polymer chains between crosslinks^28^. As the network structure heavily depends on the crosslinking process for which same components were used (and constant mixing ratio thereof) we believe that the rate of network formation process determined by employed curing temperature substantially influences final networks topology in terms of network strand length and fraction of defects^29,30^. However, precise quantification of these factors is beyond the scope of the present investigation. Nevertheless, in order to determine cross-linking density a result from thermodynamics relating elastic tensile modulus, *E*, to cross-linking density, *ρ*_K_, was employed

4 $$ E = \frac{3}{2}kT\rho_{K} , $$

where *k* is Boltzman constant and *T* absolute temperature^4^.

To better compare differences in *G*′ for various curing temperatures the limiting values *G′*_∞_ (obtained from curing reaction fitting) were normalised to *G′*_∞_ of the sample cured at 150 °C (see the last column in Table 3). Thus, one can see that at 120 °C and 100 °C mechanical properties drop to approximately 80% and 75%, respectively. This clearly demonstrates that the usage of higher curing temperature yields polymer network with higher crosslinking density than the one obtained at lower curing temperatures.

Curing temperature effect on final mechanical properties of Sylgard 184 were supported by the results of tensile test as well. Even though tensile test provides three main mechanical characteristics, namely *E*, strength at break and elongation at break, we have decided to focus mainly on elastic modulus as it is the most robust out of the three and least sensitive to sample structural imperfection (e.g. cracks and defects).

**Figure 3:**
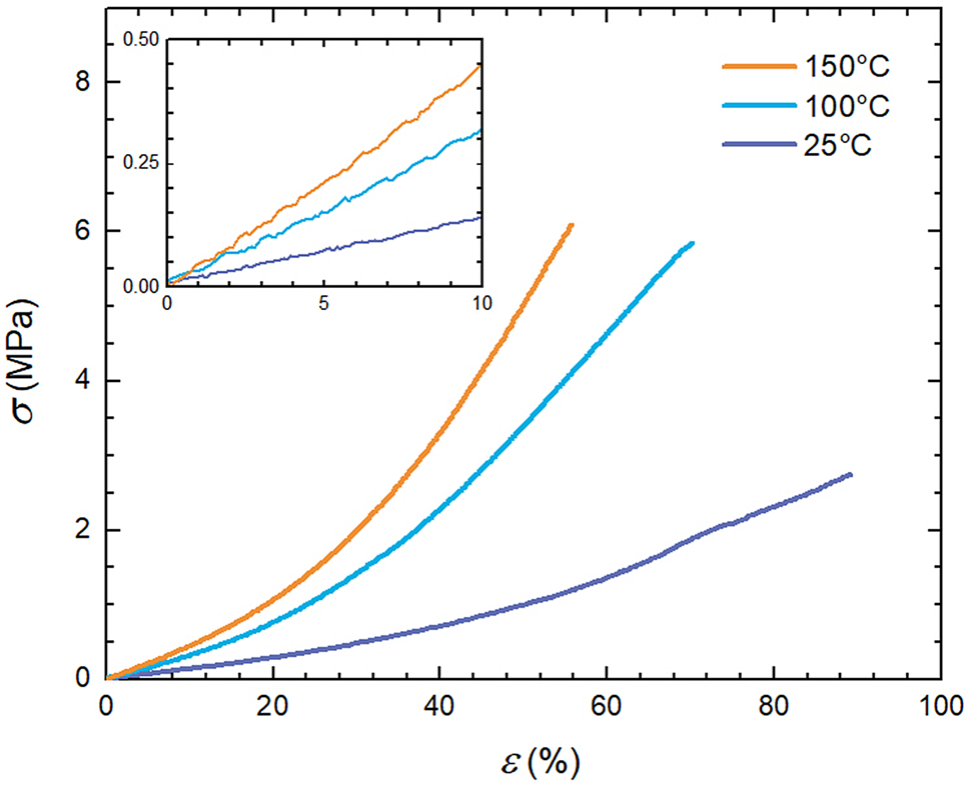
Tensile test curves of Sylgard 184 cured at different temperatures.

Recorded stress–strain curves exhibit shape typical for elastic materials, i.e. convexly bent curve with very short linear Hook’s region (Fig. 3), which however still enables one to determine *E* given as a slope of the line within 0–10% strain range.

**Figure 4:**
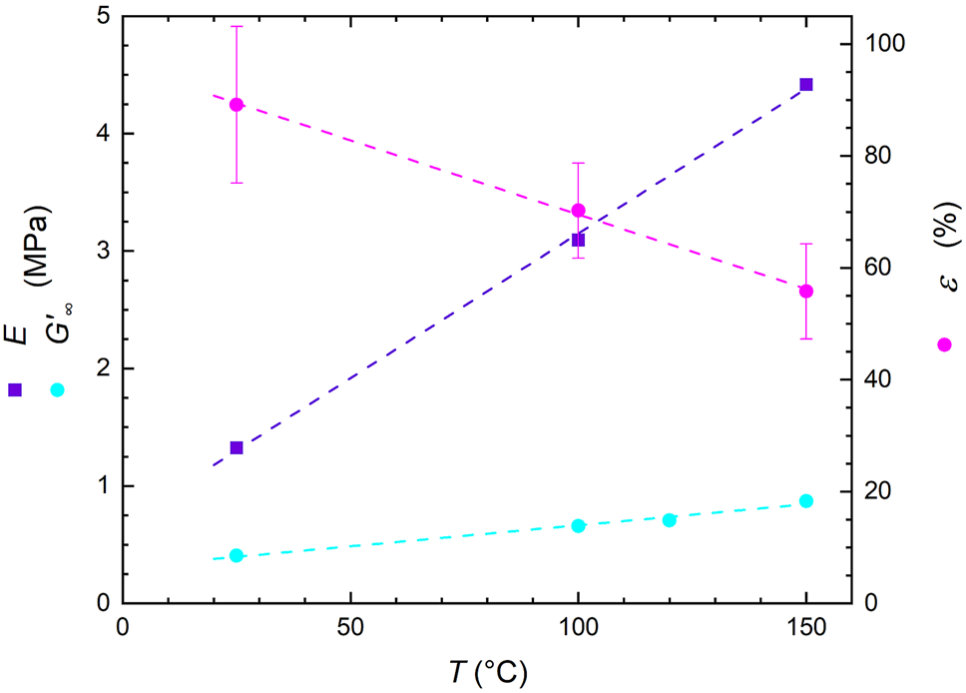
Impact of curing temperature on mechanical properties of neat Sylgard 184.

It can be clearly seen that elevated curing temperature, on one hand leads to higher modulus and strength, even though the increase of strength does not follow linear behaviour as in the case of *E*, but on the other hand it results in linear drop in strain at break (Fig. 4; average values from 5 measurements).

This experimental finding suggests that curing temperature does not only affect rate of curing reaction however it also influences qualitatively the properties of the final polymer network, which is consequently translated to different mechanical properties. The fact that higher curing temperature yields substantially stronger (*E* increases from 1 to 4 MPa for samples cured at 20 °C and 150 °C, respectively; see Table 4) but less extensible material hints at the fact that curing temperature has a strong bearing on the crosslinking density of Sylgard’s polymer network. Cross-linking density calculated using Eq. (4) yields approximately 10^26^ cross-links per cubic meter and the crosslinking density increases linearly with curing temperature (Table 4) confirming that elevated temperatures result in the polymer network with higher crosslinking density compared to analogues prepared at lower temperatures.

**Table 4:** Cross-linking density.

Curing temperature (°C)	*E* (MPa)	*ρ*_K_ (m^–3^)
25	1.32	2.1 × 10^26^
100	3.09	5.0 × 10^26^
150	4.42	7.2 × 10^26^

This seems logical enough as there are no other factors which could have so considerable impact on the mechanical properties of the final material.

The modulus versus temperature trend found for *E* was also measured and confirmed for *G*′ (Fig. 4). Here, *G*′ follows also increases with temperature, nevertheless at a slower rate owing to different deformation modes, i.e. elongation versus shear, and their sensitivity to polymer network crosslinking density.

### Silicone oil-extended Sylgard 184

Extension of Sylgard 184 with silicone oil, through mixing it in the given concentration, is carried out in order to soften the elastomer, by which we particularly denote decreasing *E* and *G*′.

**Figure 5:**
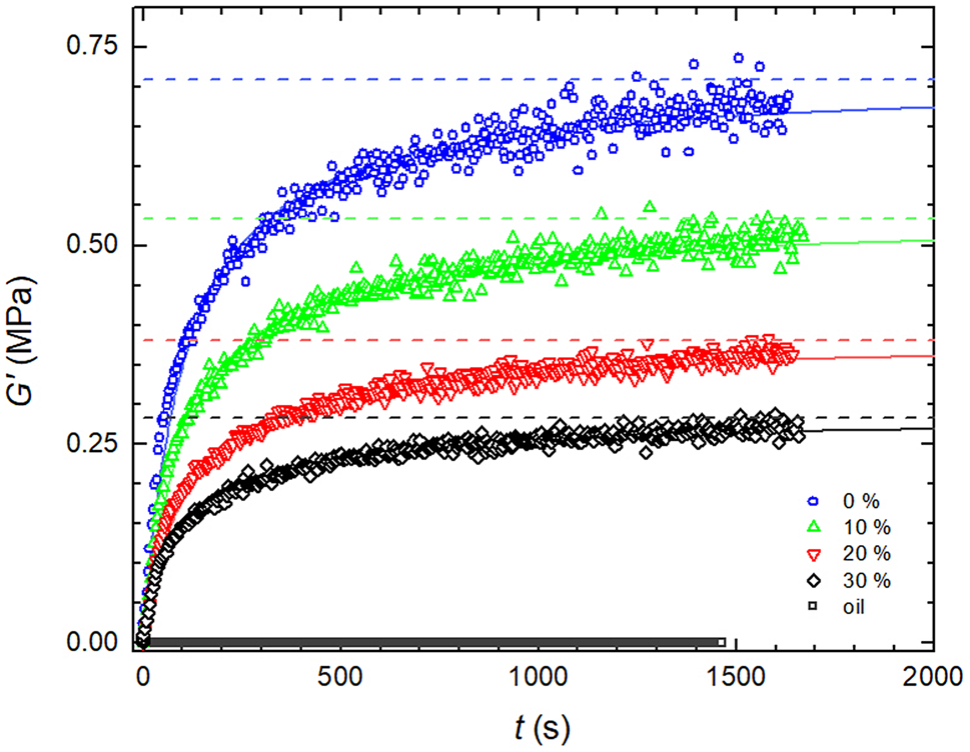
The effect of oil addition on curing kinetics and conversion values of Sylgard 184 (cured at 120 °C).

Addition of silicone oil does not introduce a competitive component which would consume Sylgard’s curing agent and thus negatively affect the curing system of Sylgard 184. This was confirmed by a test in which viscosity of silicone oil with Sylgard’s curing agent added was measured in time. Results showed no viscosity increase which would otherwise indicate reaction between silicone oil and the curing agent (Fig. 5).

**Figure 6:**
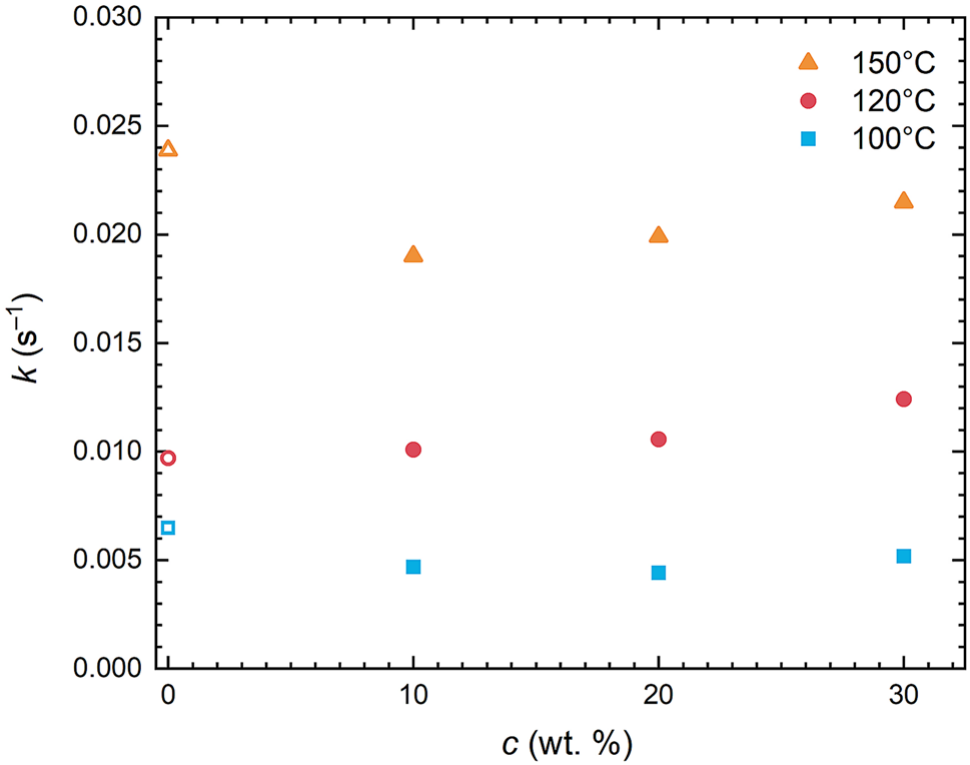
Dependence of rate constant, *k*, on curing temperature and oil content, c.

As far as the reaction kinetics is concerned its character does not change the presence of silicone oil in the reaction mixture—time evolution of *G*′ is still very well described by the same second order kinetics model (Eq. 3, Fig. 5). This is reflected also in the value of rate constant, *k*, which apart from minor fluctuations around a mean value for a particular temperature does not show any other clear trend with added silicone oil (Fig. 6). However as shown in Fig. 6 *k* strongly depends on curing temperature rising from 0.004 to 0.017 s^–1^ for temperatures 100 °C and 150 °C, respectively, accelerating the kinetics more than four times.

**Figure 7:**
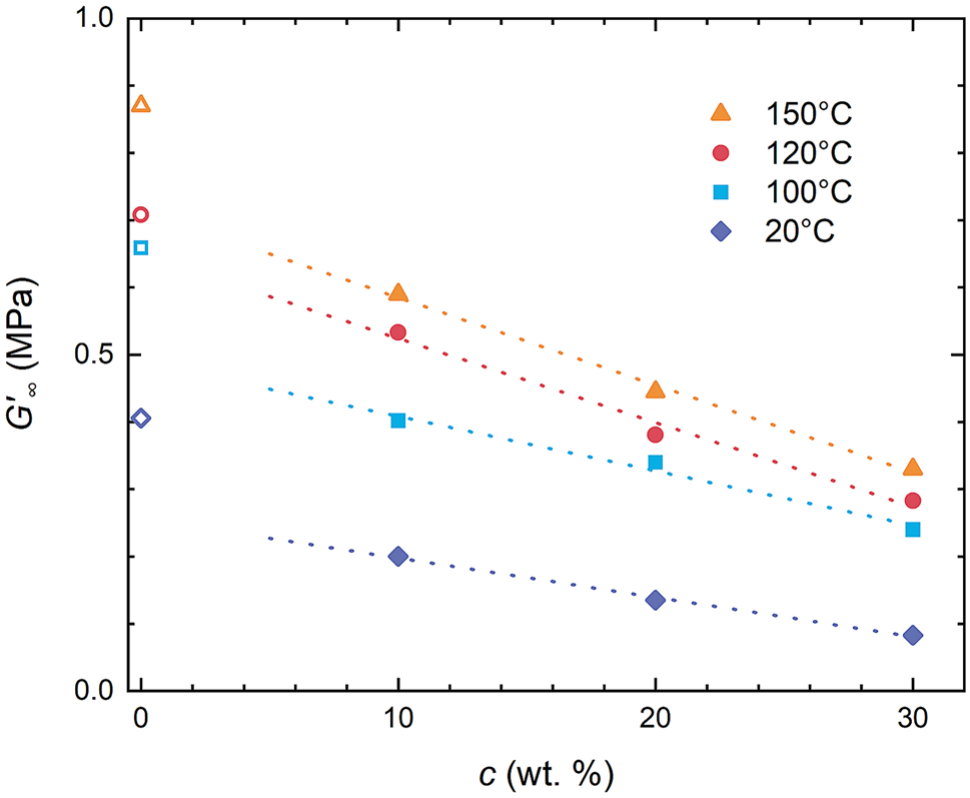
Crosslinking density decrease with oil content for different temperatures derived from rheological measurements.

Despite the fact that kinetics virtually stays the same regardless the added oil, fully cured shear modulus (*G′*_∞_) has seen a significant decrease with silicone oil gradually dropping positions of *G′*_∞_ asymptotes for 120 °C (Fig. 5) and for all investigated temperatures in the comprehensive plot showing *G′*_∞_ versus oil concentration (Fig. 7). Therein an initial drop of *G*′ is followed by a linear decent with oil added.

**Figure 8:**
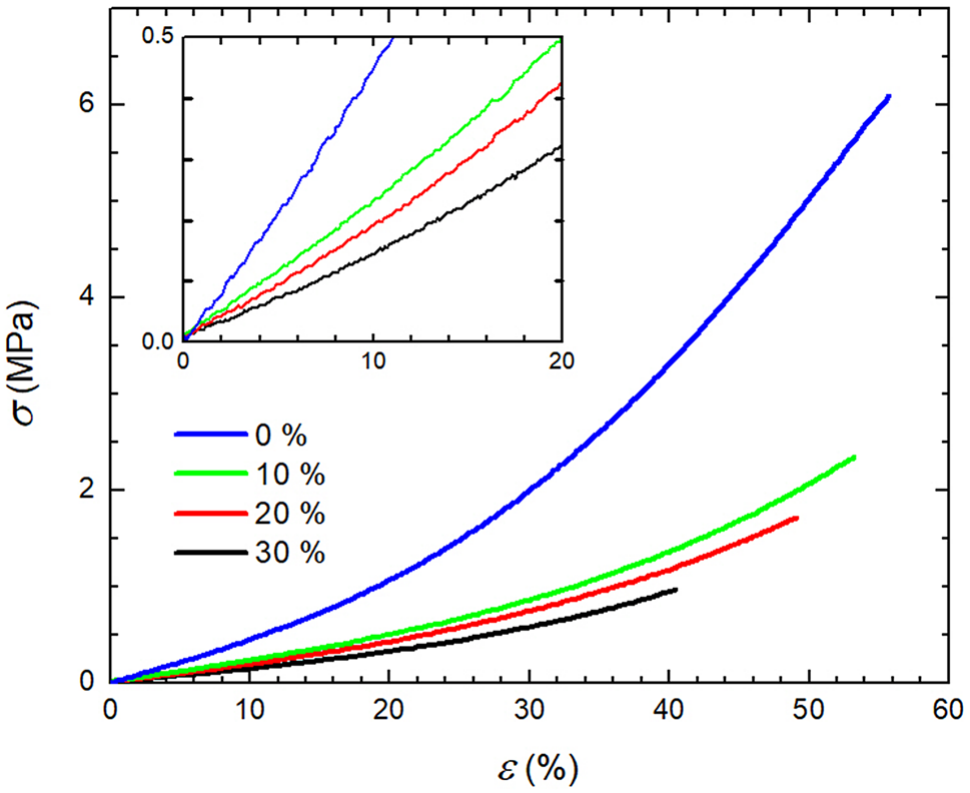
Tensile test curves for Sylgard 184 with added oil (cured @150 °C).

Typical stress–strain curve for Sylgard 184 extended with silicone oil is illustrated in Fig. 8 for the set of samples cured at 150 °C, however similar record was obtained even for other temperatures.

As can be seen, the character of samples containing silicone oil resembles very closely that of the pure elastomer (blue symbols) with typical concavely shaped curve, which monotonously increases with strain up to the break. Although both the maximal *σ* and corresponding *ε* tend to decrease with oil loading of the extended elastomeric system from about 2–0.8 MPa and 54–40%, respectively, the slope at the beginning of curve marking the *E* seems again more robust criterium for determination of mechanical properties and their variation with silicone oil concentration.

**Figure 9:**
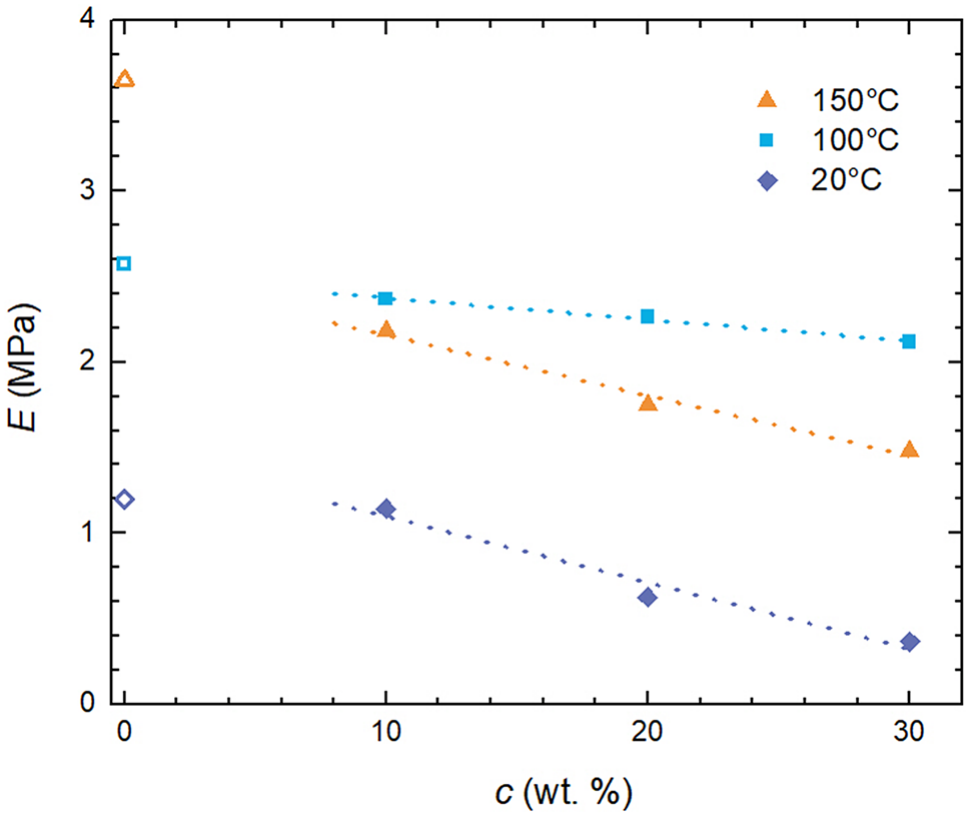
Impact of oil extension of Sylgard 184 on tensile modulus for various temperatures.

Therefore, values of *E* were used to track the impact of oil on mechanical properties of Sylgard 184. Figure 9 shows how *E* progresses with silicone oil added to standard reaction mixture for three tested curing temperatures. As can be seen for all temperatures *E* decreases with oil concentration and while this decrease is approximately linear in the case of 25 °C and 100 °C, the highest investigated curing temperature of 150 °C sees a substantial drop of *E* from 3.7 to 2.2 MPa with 10 wt.% of oil added, which is subsequently followed by a further mild descent with oil content. Although generally it holds that the higher the curing temperature the higher the *E* (or even more generally mechanical properties) for 150 °C the oil extension leads to decrease of *E* below values obtained/measured for samples cured at 120 °C. This may suggest that combination of rapid curing with present silicone oil may lead to lower network crosslinking density or even some cracks/defects in the network, which consequently causes lowering of elongation at break. One way or another it seems that oil extension results in non-monotonous (exhibiting temperature related maximum) dependence of mechanical properties unlike in the case of pure Sylgard 184.

## Conclusions

Recorded increase in mechanical properties with curing temperature is caused by increased crosslinking density calculated to be of the order of 10^26^ m^–3^. As the only parameter which differed during preparation was the curing temperature, we believe that it has a significant impact not only on time, during which Sylgard 184 can be cured, but also on type of network formation/crosslinking through affecting the reaction kinetics of curing. Curing at elevated temperatures leads to networks featuring higher density of nodes which in turn positively affects mechanical properties. On the other hand, curing carried out at low temperatures (including ambient temperatures) leads to looser polymer networks in terms of their crosslinking and consequently lower moduli (elongation, shear) and strength however slightly higher strain/elongation at break.

Extension of Sylgard 184 with silicone oil, while not having an impact on the curing process (with rate constant *k* not significantly affected), substantially softens the silicone elastomer through lowering mechanical properties of the final network. As silicone oil was found not to interact with either component of Sylgard 184 precursors, its role lies in spatial/steric factors further decreasing the crosslinking density of the final polymer network. Thus, within a reasonable concentration range, one can easily prepare a soft silicone elastomer system/binder/matrix with tuneable mechanical properties, namely modulus, while keeping all the other advantages of Sylgard 184 (e.g. chemical inertness, transparency).

These materials can find their use in a number of applications where tunability of mechanical properties is crucial, ranging from MEMS over magnetorheological elastomers (MRE) to biomaterials.

## Data Availability

The data that support the findings of this study are available from the corresponding author, M. S., upon reasonable request.
